# Genetic interactions among *Brca1*, *Brca2*, *Palb2*, and *Trp53* in mammary tumor development

**DOI:** 10.1038/s41523-021-00253-5

**Published:** 2021-04-23

**Authors:** Yanying Huo, Pier Selenica, Amar H. Mahdi, Fresia Pareja, Kelly Kyker-Snowman, Ying Chen, Rahul Kumar, Arnaud Da Cruz Paula, Thais Basili, David N. Brown, Xin Pei, Nadeem Riaz, Yongmei Tan, Yu-Xiu Huang, Tao Li, Nicola J. Barnard, Jorge S. Reis-Filho, Britta Weigelt, Bing Xia

**Affiliations:** 1 Rutgers Cancer Institute of New Jersey, New Brunswick, NJ USA; 2 Department of Radiation Oncology, Rutgers Robert Wood Johnson Medical School, New Brunswick, NJ USA; 3 Department of Pathology, Memorial Sloan Kettering Cancer Center, New York, NY USA; 4 Department of Radiation Oncology, Memorial Sloan Kettering Cancer Center, New York, NY USA; 5 Stomatological Hospital of Guangzhou Medical University, Guangzhou, China; 6 The First Affiliated Hospital of Fujian Medical University, Fuzhou, China; 7 Department of Pathology and Laboratory Medicine, Rutgers Robert Wood Johnson Medical School, New Brunswick, NJ USA; 8 Present Address: Department of Physiology, College of Medicine, Al-Mustansiriyah University, Baghdad, Iraq; 9 Present Address: Centre for Brain Research, Indian Institute of Science (IISc), Bangalore, India

**Keywords:** Cancer genetics, Epistasis

## Abstract

Inherited mutations in *BRCA1*, *BRCA2*, and *PALB2* cause a high risk of breast cancer. Here, we conducted parallel conditional knockout (CKO) of *Brca1*, *Palb2*, and *Brca2*, individually and in combination, along with one copy of *Trp53*, in the mammary gland of nulliparous female mice. We observed a functional equivalence of the three genes in their basic tumor-suppressive activity, a linear epistasis of *Palb2* and *Brca2*, but complementary roles of *Brca1* and *Palb2* in mammary tumor suppression, as combined ablation of either *Palb2* or *Brca2* with *Brca1* led to delayed tumor formation. Whole-exome sequencing (WES) revealed both similarities and differences between *Brca1* and *Palb2* or *Brca2* null tumors. Analyses of mouse mammary glands and cultured human cells showed that combined loss of BRCA1 and PALB2 led to high levels of reactive oxygen species (ROS) and increased apoptosis, implicating oxidative stress in the delayed tumor development in *Brca1*;*Palb2* double CKO mice. The functional complementarity between BRCA1 and PALB2/BRCA2 and the role of ROS in tumorigenesis require further investigation.

## Introduction

Monoallelic germline mutations in *BRCA1* and *BRCA2* cause high risks of breast and ovarian cancer and also increase the risk of pancreatic and other cancers^1,2^. The two genes encode large proteins with no sequence similarity but share critical functions in the DNA damage response, such as the repair of DNA double-strand breaks (DSBs) by homologous recombination (HR), protection of stalled DNA replication forks, and DNA damage-induced cell cycle checkpoints^3,4^. As such, the two proteins ensure faithful DNA replication, DNA repair, and chromosomal separation, serving as “chromosome custodians” to suppress tumor development^5^. Other than *BRCA1* and *BRCA2*, inherited mutations in about a dozen other genes are also associated with increased breast cancer risk^6,7^. Among these is *PALB2*, which encodes a major BRCA2 binding partner that controls its intranuclear localization and stability^8^ and links BRCA1 and BRCA2 in HR repair and DNA damage-induced cell cycle checkpoint response^8–11^. Consistent with its similar molecular functions to BRCA1 and BRCA2, monoallelic germline mutations in *PALB2* also confer a high risk of breast cancer and increase the risk of ovarian and pancreatic cancers^7,12^. Our group and others have observed that biallelic pathogenic alterations in HR DNA repair-related genes, including *BRCA1* and *BRCA2*, are prevalent across many malignancies, are often associated with genomic features of HR deficiency (HRD), and that in ovarian, breast, and prostate cancers, these biallelic alterations are mutually exclusive of each other^13–15^.

Many mouse models have been generated to study the function of BRCA1, BRCA2, and PALB2 in development and tumor suppression. Germline knockout of each gene caused embryonic lethality rather than tumor predisposition^16–19^. This has generally been attributed to their critical role in HR, which is required for DNA replication and proliferation of normal cells. Conditional knockout (CKO) of each gene in the mammary epithelium led to tumor development with long median latencies ~1.5 years^19–24^. Whenever tested, mutations in *Trp53* were found in most of the tumors^20,21,23^. Co-ablation of *Trp53* with each of the three genes led to more efficient mammary tumor formation^19,23,25,26^, and a *Trp53* heterozygous background also promoted mammary tumorigenesis in *Brca1* CKO mice^22,24^. Collectively, the above findings suggest that p53 is a strong barrier to tumor development following the loss of the BRCA and PALB2 proteins in mammary epithelial cells (MECs).

Although various CKO models have been generated for BRCA1/2- and PALB2-associated mammary tumorigenesis, the studies were conducted separately for each gene, using different Cre drivers and in different genetic backgrounds. Also, the genetic interactions among the genes in cancer development have not been determined. In this study, we ablated, in parallel, each of the three genes along with one copy of *Trp53* in the mouse mammary gland. This allowed us to compare directly the latency and penetrance of tumor development associated with each gene and the histopathological and genomic features of the mutant tumors in the same setting. Combined ablations of the genes further allowed us to assess the genetic relationships among them in the context of tumor development. Moreover, we studied the impact of combined loss of BRCA1 and PALB2 in both mouse mammary glands and cultured human cells. Our results revealed new insights into the mechanisms of hereditary breast cancer development.

## Results

### Tumor development in mice with individual and combined ablations of *Brca1*, *Palb2*, *Brca2*, and *Trp53*

To compare directly the latency, penetrance, and various features of BRCA1, BRCA2, and PALB2-associated mammary tumor development, we set out to ablate the three genes in parallel in mice using *Wap-cre*, which is predominantly expressed in the secretory epithelium in the mammary gland^27^. To facilitate mammary tumor formation, we chose to co-delete one copy of *Trp53*. As the floxed alleles (*Brca1*^*f5-13*^, *Brca2*^*f11*^, *Palb2*^*f2-3*^, and *Trp53*^*f2-10*^) and the *Wap-cre* allele were from different genetic backgrounds, with elements of C57BL/6, 129sv, and FVB-N, we first conducted a multi-step crossing of the source mice to generate several “common ancestor” mice carrying all five alleles. A single pair of the ancestor mice were then intercrossed, and the subsequent allele separation gave rise to mice with different combinations of the alleles. Further breeding allowed us to establish cohorts of the following genotypes: *Trp53*^*f/w*^;*Wap-cre*, *Brca1*^*f/f*^;*Trp53*^*f/w*^;*Wap-cre*, *Palb2*^*f/f*^;*Trp53*^*f/w*^;*Wap-cre*, *Brca2*^*f/f*^;*Trp53*^*f/w*^;*Wap-cre*, *Brca1*^*f/f*^;*Palb2*^*f/f*^;*Trp53*^*f/w*^;*Wap-cre*, *Brca1*^*f/f*^;*Brca2*^*f/f*^;*Trp53*^*f/w*^;*Wap-cre*, and *Palb2*^*f/f*^;*Brca2*^*f/f*^;*Trp53*^*f/w*^;*Wap-cre*. For simplicity, these mice will be referred to as control, *B1p53*, *P2p53*, *B2p53*, *B1P2p53*, *B1B2p53*, and *P2B2p53* mice, respectively, hereafter. Note that despite the above effort to equalize the genetic backgrounds of the mice in different groups, the backgrounds were still mixed and remained a potential confounding factor for data interpretation.

As the *Wap* promoter is responsive to female hormones and is most active during late pregnancy and lactation, *Wap-cre* model mice are routinely mated to induce pregnancy and lactation and therefore maximum gene deletion^20,27^. However, efficient mammary tumor formation has been reported in nulliparous *Brca1*^*f11/f11*^;*Trp53*^*f5-6/f5-6*^;*Wap-cre* mice^28^. To avoid the scenario where the subsequent mammary gland involution after weaning might cause unequal death of cells with different gene deletions complicating data interpretation, we omitted the mating step and monitored nulliparous females for their entire life span.

**Fig. 1:**
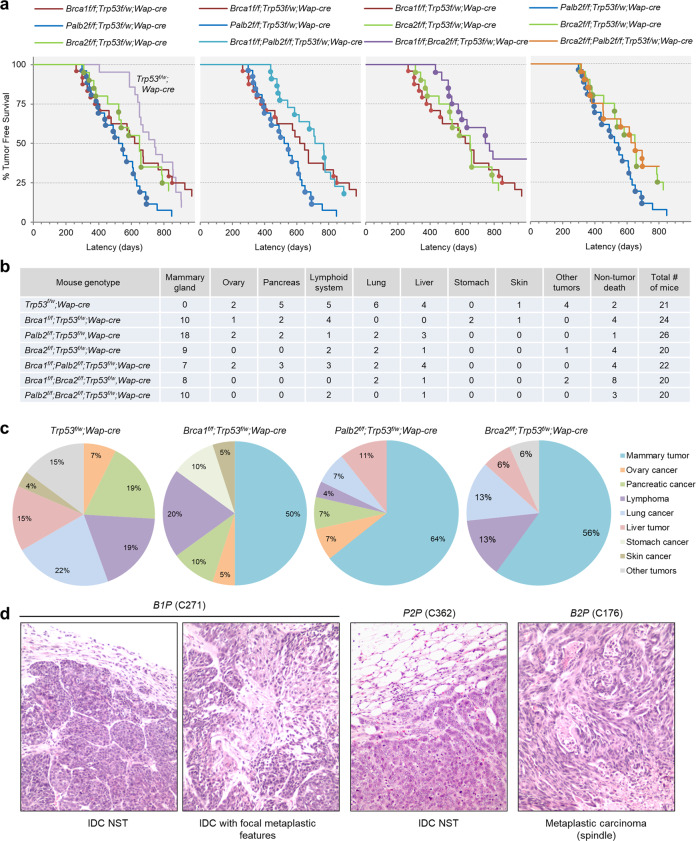
Tumor development in mice with individual and combined ablations of *Brca1*, *Palb2*, *Brca2*, and *Trp53*. **a** Tumor-free survival curves of model mice with indicated genotypes. Mammary tumors are denoted by filled cycles. **b** Summary of tumor types and the numbers of mice of different genotypes affected by each tumor type. Note that some mice developed multiple tumors of either the same or different types. **c** Tumor spectra of mice with different genotypes. **d** Representative micrographs of H&E-stained tissue sections of mammary tumors from mice with indicated genotypes. IDC NST invasive ductal carcinoma of no special type.

At around 300 days of age, *B1p53*, *P2p53*, and *B2p53* mice all began to bear tumors (Fig. 1a). From this point to ~500 days of age, all but one tumor that formed in these three groups of mice were mammary tumors. Afterward, *P2p53* mice continued to develop mammary tumors at a similar pace, *B1p53* mice showed a long gap in time before developing additional mammary tumors, while *B2p53* mice showed a gap in not only mammary tumor but also overall tumor development, before resuming tumor formation at a pace largely parallel to that of *P2p53* mice (Fig. 1a and Supplementary Fig. 1a). The biphasic kinetics of mammary tumor development in *B1p53* mice, or both mammary and overall tumor development in *B2p53* mice, was possibly due to stochastic and tumor-suppressive genetic or epigenetic events that were more common in these two groups of mice in comparison with *P2p53* mice. Beside mammary tumors, the mice developed tumors in a variety of tissues, such as the lymphoid system (mostly thymus), pancreas, ovary, and liver, etc. (Fig. 1b and Supplementary Data 1). Overall, 50%, 64%, and 56% of tumors that developed in *B1p53*, *P2p53*, and *B2p53* mice, respectively, were mammary tumors (Fig. 1c). The nearly identical timing of the “first wave” of mammary tumor development suggests that BRCA1, BRCA2, and PALB2 may be functionally equivalent in mammary tumor suppression. The fact that both mammary and overall tumor development were most efficient in *P2p53* mice also implies that PALB2 is at least as critical a tumor suppressor as BRCA1 and BRCA2.

In comparison, control mice not only showed a longer tumor latency than all the above three groups of mice but also, strikingly, failed to develop any mammary tumor. Instead, these mice developed lymphomas and tumors in the lung, pancreas, liver, and ovary, etc. (Fig. 1b, c and Supplementary Data 1). In all four groups, in most cases one tumor was found per mouse; however, a significant number of mice developed multiple tumors, which was most common in the control group (Supplementary Data 1). The fact that the control mice did not develop any mammary tumor while 50% or more of *B1p53*, *P2p53*, and *B2p53* mice did clearly demonstrate the tumor-suppressive activities of BRCA1, PALB2, and BRCA2 in the mammary gland. At the same time, the findings suggest that the *Wap-cre* used may have “leaky” expression in other tissues and the development of non-mammary tumors might be due to unintended gene deletion therein. Indeed, various degrees of recombination of the foxed *Trp53*, *Brca1*, and *Palb2* alleles were detected by PCR genotyping in available non-mammary tumors (Supplementary Fig. 2), indicating that the *Wap-cre* was indeed leaky and that at least some of those tumors might stem from the unintended gene ablation events. For example, one of the liver tumors arising from *P2p53* mice showed complete *Palb2* deletion and likely stemmed from PALB2 loss; a lung tumor and a liver tumor from *B1p53* mice showed strong *Brca1* deletion to a degree similar to that found in mammary tumors from mice in the same cohort (Supplementary Fig. 2), suggesting that these two tumors might have arisen due to BRCA1 loss. On the other hand, tumors showing no clear recombination of any of the floxed genes are likely unrelated to the floxed genes, and the etiology of tumors with overall limited deletions of *Trp53*, *Brca1*, or *Palb2* cannot be assigned without detailed analysis of tumor purity and other characteristics.

Interestingly, initial mammary tumor development in *B1P2p53* mice was markedly delayed in comparison with that in either *B1p53* or *P2p53* mice (Fig. 1a and Supplementary Fig. 1b). Similarly, *B1B2p53* mice also showed a delay in the initial mammary tumor development in comparison with either *B1p53* or *B2p53* mice (Fig. 1a and Supplementary Fig. 1c). However, except for the difference between *B1P2p53* and *P2p53* groups, the differences between other pairs were not statistically significant due to the above-noted gap in *B1p53* mice or the biphasic tumor development in *B2p53* mice. Notably, *P2B2p53* mice showed similar kinetics of tumor development to that of *B2p53* mice (Fig. 1a and Supplementary Fig. 1d), and tumor-free survivals of *B1P2p53* and *B1B2p53* mice were also very similar (Fig. 1a and Supplementary Fig. 1e). This epistatic relationship between *Brca2* and *Palb2* lends further support to the notion that the two gene products function as a complex in tumor suppression, whereas the delayed tumor formation in *B1P2p53* and *B1B2p53* mice suggests that BRCA1 and the PALB2/BRCA2 complex not only share similar fundamental tumor-suppressive activity but also possess mutually independent functions that influence the progression of the tumorigenic process, possibly by impacting cell fitness.

Histopathologic analyses were conducted to evaluate the characteristics of the mammary tumors arising in the model mice. The tumors the engineered mice developed were high-grade invasive ductal carcinomas of no special type (IDC NST) or tumors with metaplastic elements, including spindle morphology, chondroid metaplasia, and squamous differentiation (Fig. 1d and Table 1), regardless of the genotypes of the source mice. Overall, the histologic features of tumors were similar across the different genotypes and largely resembled those of human *BRCA1/2* and *PALB2* breast cancers, with the only notable difference being that tumors in *P2B2p53* mice appeared to be more uniform and showed a virtual absence of metaplastic features.

**Table 1:** Summary of mammary tumors analyzed in this study.

Case	Genotype	Latency	Histology	Chr14	*Met*	Mut #	LST	NtAI
XB02	B1P	263	IDC w/focal metaplastic features	D*	–	32	27	10
XB47	B1P	301	IDC NST	D	–	56	26	14
XB01	B1P	331	IDC w/focal metaplastic features	D*	–	35	33	13
XB31	B1P	350	IDC NST	–	–	64	25	11
XB04	B1P	378	IDC w/focal metaplastic features	D	–	28	20	11
XB10	P2P	298	Metaplastic carcinoma (spindle)	D	A	51	21	12
XB07	P2P	395	Metaplastic carcinoma (spindle)	D	A	33	19	9
XB32	P2P	489	IDC NST	–	–	58	12	10
XB08	P2P	571	IDC NST	D*	–	44	26	14
XB33	P2P	652	IDC NST	D*	A	100	17	12
XB34	B2P	342	Metaplastic carcinoma (spindle)	–	A	45	27	11
XB13	B2P	382	Metaplastic carcinoma (squamous)	D*	–	152	15	8
XB30	B2P	386	IDC NST	D*	A	33	22	12
XB11	B2P	583	IDC NST	–	–	35	23	9
XB35	B2P	785	IDC w/focal metaplastic features	D*	–	78	17	9
XB19	B1P2P	437	IDC w/focal metaplastic features	–	–	35	32	18
XB22	B1P2P	442	IDC w/focal metaplastic features	D*	–	36	30	16
XB21	B1P2P	477	IDC NST	–	–	47	18	14
XB39	B1P2P	586	IDC NST	–	–	50	24	10
XB23	B1P2P	679	IDC NST	–	–	45	33	12
XB36	B1B2P	434	Metaplastic carcinoma (spindle)	–	–	73	28	15
XB17	B1B2P	517	IDC w/focal metaplastic features	–	–	63	26	15
XB37	B1B2P	536	Metaplastic carcinoma (squamous)	D*	–	51	28	13
XB38	B1B2P	577	Metaplastic carcinoma (spindle&chondroid)	D*	–	59	25	16
XB16	B1B2P	629	IDC NST	D*	–	89	24	9
XB26	P2B2P	334	IDC NST	D*	A	22	19	11
XB27	P2B2P	358	IDC NST	D	A	26	18	10
XB41	P2B2P	651	IDC NST	–	–	59	24	10
XB48	P2B2P	694	IDC NST	D*	–	54	28	11
XB49	P2B2P	696	IDC NST	D*	–	68	23	15
XB42	p53KO	324	IDC w/focal metaplastic features	–	A	28	17	10
XB44	p53KO	373	Metaplastic carcinoma (spindle)	–	–	47	13	11
XB43	p53KO	426	IDC NST	–	A	28	20	14
XB46	p53KO	436	IDC NST	–	–	26	14	10
XB45	p53KO	562	Metaplastic carcinoma (spindle)	D*	A	31	16	11

Samples in each group were sorted by latency (days).

*IDC* invasive ductal carcinoma, *IDC NST* IDC of no special type, *D* deletion or loss (asterisk denotes loss of part of the chromosome), *A* amplification, *Mut #* number of non-synonymous mutations, *LST* large-scale state transitions, *NtAI* telomeric allelic imbalance.

### Genomic analyses of the mammary tumors

**Fig. 2:**
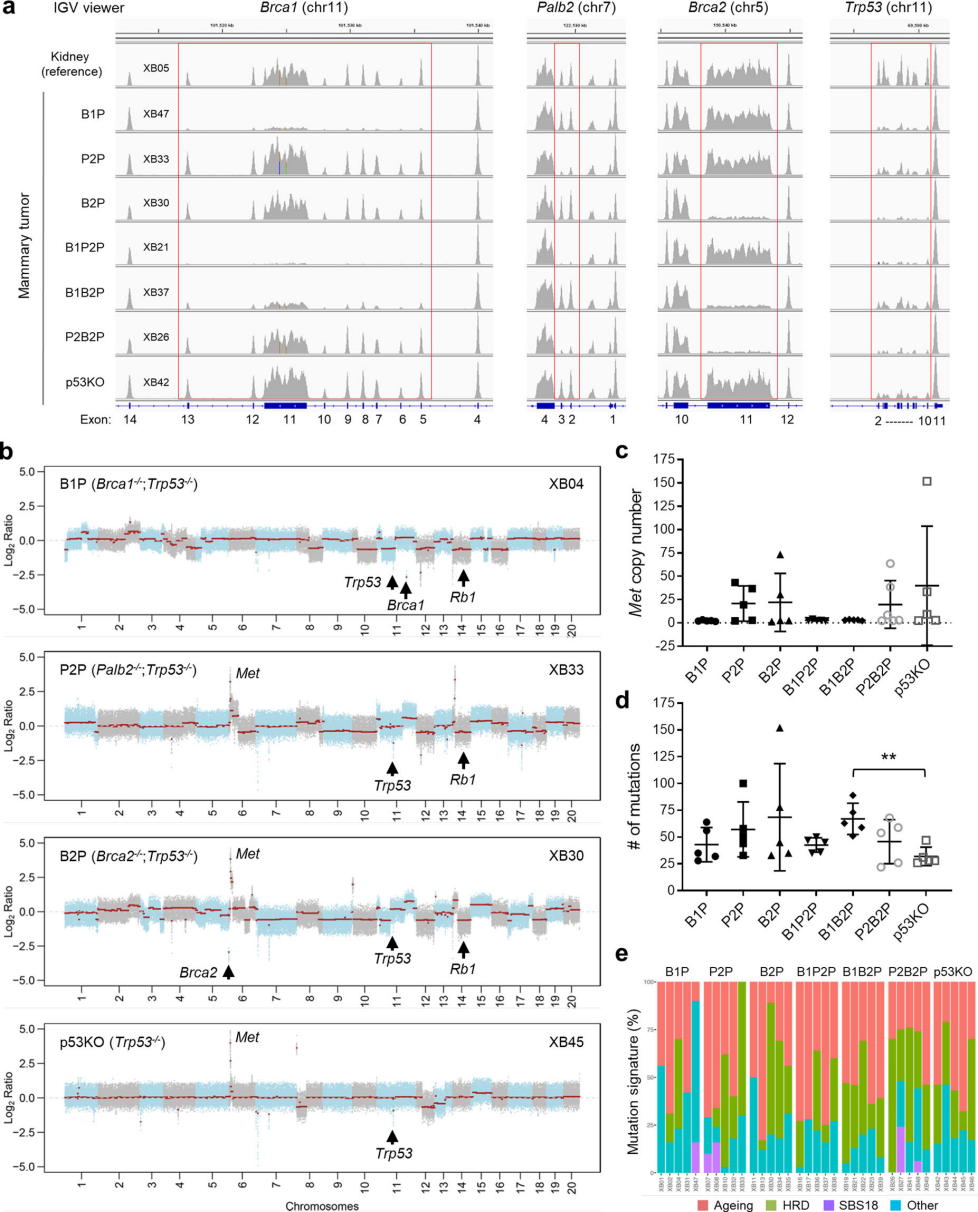
Genomics features of the mouse mammary tumors revealed by WES (I). **a** Genomic status of *Brca1*, *Palb2*, *Brca2*, and *Trp53* loci in representative mammary tumors of indicated genotypes. Images were generated with an integrated genome viewer (IGV). **b** Genome-wide copy number plots of representative mammary tumors of indicated genotypes. Copy number of *Met* (**c**), number of somatic mutations (**d**), and mutation signatures (**e**) of mammary tumors of indicated genotypes. Mutational signature decomposition was performed using DeconstructSigs. Ageing-related signatures 1 and 5, the HRD-related signature 3, and signature 18 (SBS18) were most commonly found. Other, all other mutational signatures.

To understand the genetic mechanisms of mammary tumor development in our mouse models, we conducted whole-exome sequencing (WES) of five mammary tumors of each genotype (Table 1). The results showed that intended deletions in *Brca1*, *Palb2*, *Brca2*, and *Trp53* occurred in all tumors (Fig. 2a and Supplementary Data 1), which was further confirmed by PCR-genotyping of additional tumors (Supplementary Fig. 2). Importantly, in addition to the original floxed *Trp53* allele, which was deleted by Cre, the wild-type (wt) *Trp53* allele was also invariably lost in all tumors subjected to WES (Fig. 2a and Supplementary Data 1), indicating that the loss of p53 function is a prerequisite for mammary tumor development in mice following the inactivation of BRCA1/2 and PALB2. As the tumors had a biallelic loss of p53, they are referred to as “B1P”, “P2P”, “B2P”, “B1P2P”, “B1B2P”, and “P2B2P” tumors to distinguish them from their source mice. For a reference, we also included five mammary tumors from (mated) *Trp53*^*f/f*^;*Wap-cre* mice obtained in a separate study conducted later in our lab using mice from the same colony. These tumors are referred to as “p53KO” tumors.

High levels of genomic instability as seen by copy number alterations (CNAs) were observed in most tumors subjected to WES (Fig. 2a). Recurrent losses of chromosome 14 (Fig. 2b) were detected in 60–80% of the tumors in all but the B1P2P and p53KO groups, in which only 20% (1/5) of the tumors displayed this feature (Table 1). The smallest region of overlap of the chromosome 14 losses encompassed *Rb1* (Fig. 2b, XB04 and XB30, respectively), a key tumor suppressor gene whose loss has been found to be a common feature shared by *BRCA1* deficient human breast and mouse mammary cancers^29,30^, suggesting a selection for a loss of *Rb1* or another gene in the region. The *Met* oncogene locus on chromosome 6 (Fig. 2b) was recurrently amplified in ~40–60% of the P2P, B2P, and P2B2P tumors; however, this genetic alteration was not present in any of the tumors with *Brca1* deletion analyzed in this study (Fig. 2c and Supplementary Fig. 3). Three of the five p53KO tumors sequenced also showed *Met* amplification, suggesting that it may be elicited by p53 loss. In addition, the majority of tumors in all groups showed loss of chromosome 12, often including (one copy of) the entire chromosome (Fig. 2b), the significance of which in tumorigenesis remains unclear.

The number of somatic mutations in the 30 *Brca1/2* and *Palb2* null tumors sequenced ranged from 22 to 152 (mean 54), with 16 tumors having more than 50 somatic mutations, whereas all 5 p53KO tumors contained fewer than 50 mutations (Fig. 2d). Due to the small size and/or large variations within each group, however, only the B1B2P group showed a statistically larger number of mutations than the p53KO reference group. No significantly recurrently mutated genes were detected either in each individual group or across different groups, and the numbers of mutations across the different *Brca1/2* and *Palb2* null groups were not statistically different. With respect to mutational signatures, an ageing signature was detected in all but one (P2P) tumor; an HRD signature was found in 2–5 tumors of each group, and an SBS18 (ROS-related) signature was seen in one B1P, two P2P, and two P2B2P tumors (Fig. 2e).

**Fig. 3:**
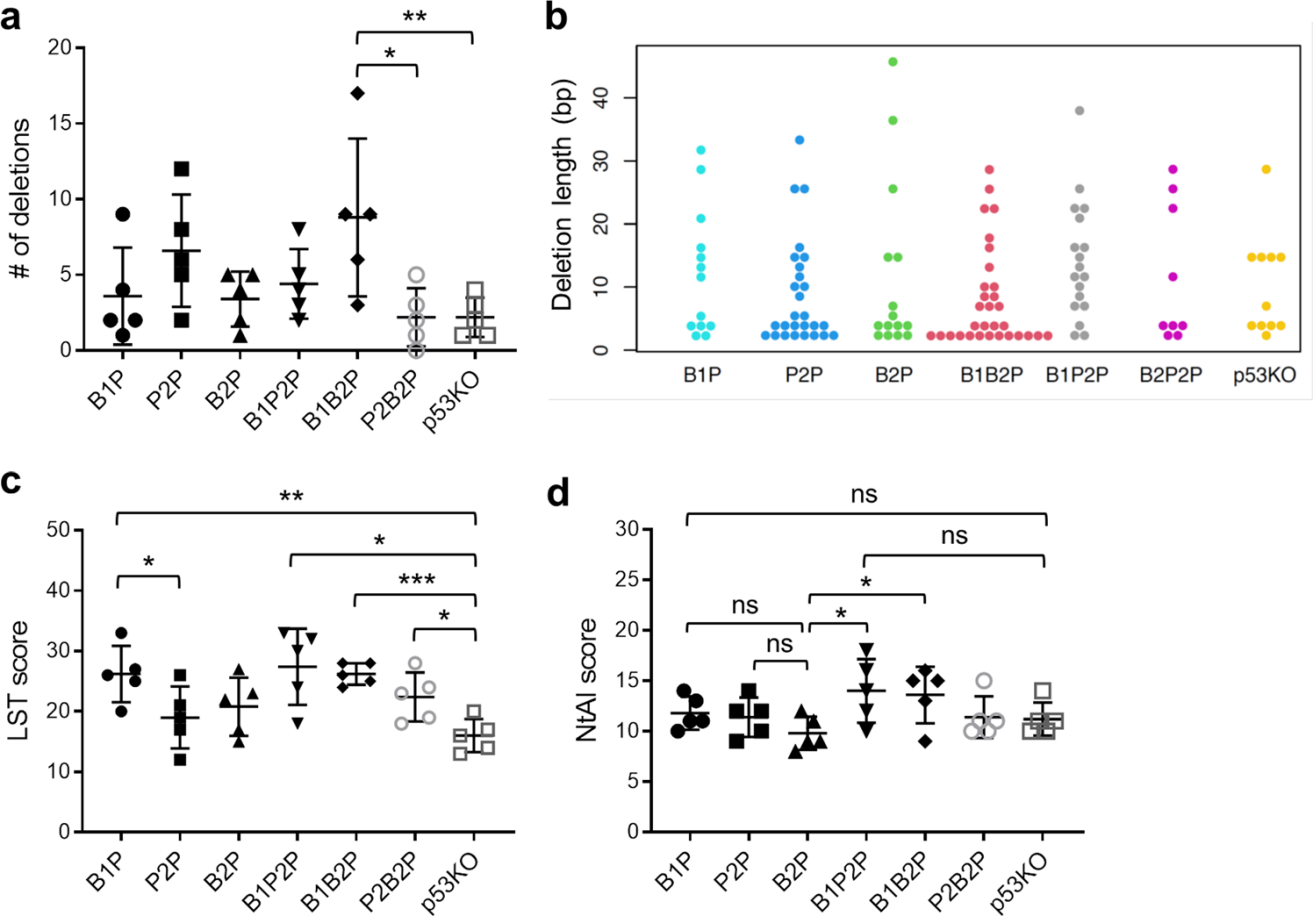
Genomics features of the mouse mammary tumors revealed by WES (II). **a** Numbers of deletions in the tumors. **b** Beeswarm plot showing indel lengths and length distributions in the tumors. **c** Large-scale state transitions (LST) scores of the tumors. **d** Telomere allelic imbalance (NTAI) score of the tumors. Error bars represent standard deviations (SD). *p < 0.05; **p < 0.01; ***p < 0.001.

We next assessed other genomics features associated with HRD in the mammary tumors. The number of small deletions and their length, which has been associated with defective HR-based repair when ≥5 bp^31^, were not statistically significantly different between the groups except that B1B2P tumor appeared to have more deletion than p53KO and P2B2P tumors (Fig. 3a, b). Large-scale state transitions (LST) scores, genomic deletions of over 10 Mb, and a genomics feature of human *BRCA1/2* mutant cancers and other cancers with HRD^32^ were higher in all *Brca1/2* and *Palb2* null tumors than in p53KO tumors (Fig. 3c), although the differences between P2P or B2P groups and the p53KO group failed to reach statistical significance. Notably, B1P tumors appeared to have higher LST scores than both P2P and B2P tumors, and both B1P2P and B1B2P tumors resembled B1P tumors, suggesting that the back-up DNA repair mechanisms utilized by cells in the absence of BRCA1 or PALB2/BRCA2 may differ and/or that the etiology of LST may involve both HR defect and loss of a specific function of BRCA1 that is not shared by PALB2 and BRCA2. Finally, we found that B1P tumors had significantly higher levels of telomeric allelic imbalance (NtAI)^33^ than B2P tumors, while P2P tumors showed an intermediate phenotype (Fig. 3d). Collectively, these findings not only underscore the critical role of BRCA1/2 and PALB2 in maintaining genome stability in the MECs but also point to a possible HR-independent function of BRCA1 in genome maintenance that is not shared by PALB2 and BRCA2, which is consistent with the different rearrangement signatures of human *BRCA1* and *BRCA2* mutant breast cancers^34^.

### DNA damage, oxidative stress, and NFκB activation in the mammary glands

**Fig. 4:**
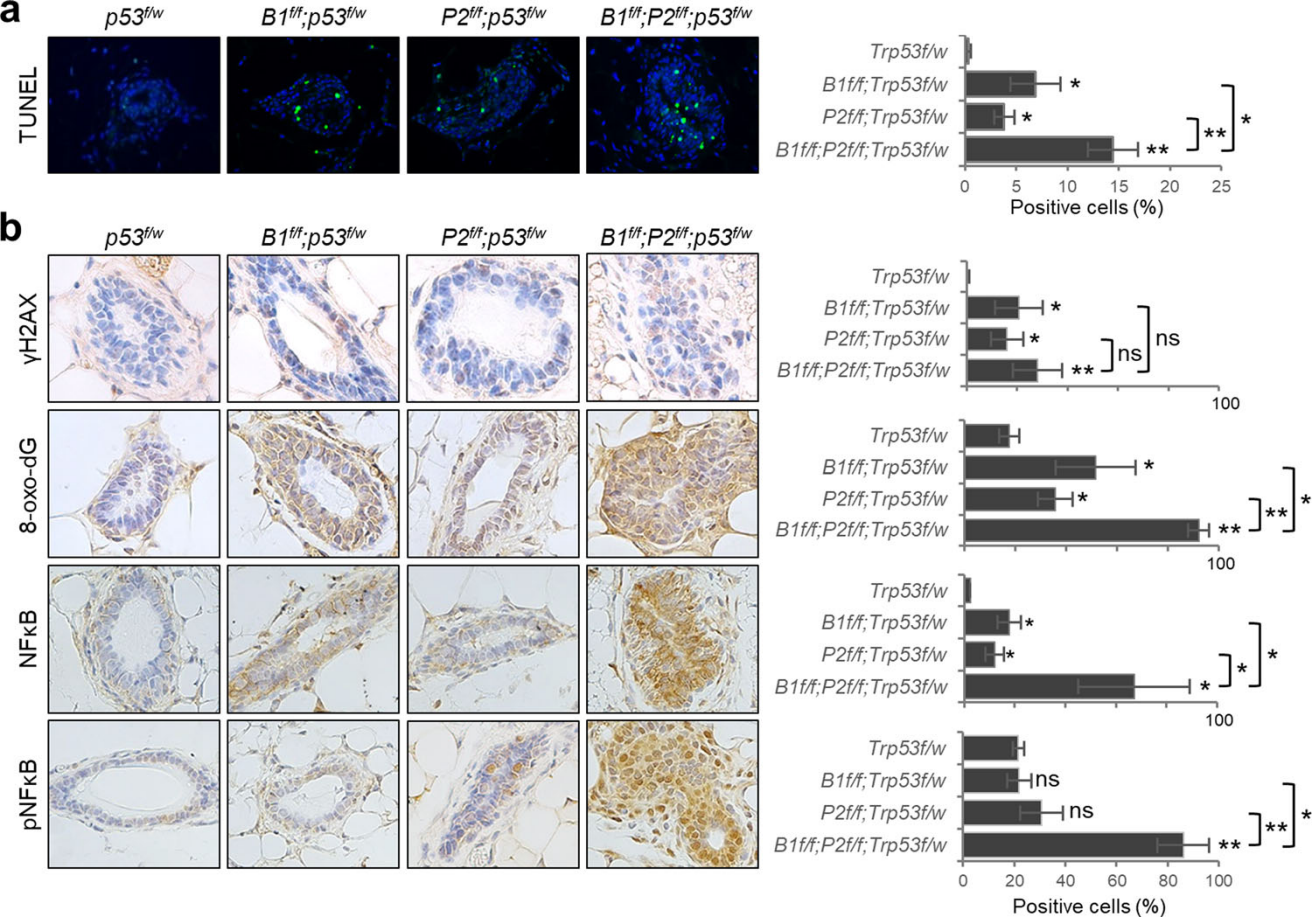
Analyses of mammary glands of 10-week-old model mice. **a** Representative images and quantifications of TUNEL signals in the mammary glands. **b** Representative IHC images and quantifications of positive cells for γH2A.X, 8-oxo-dG, NFκB p65, and phospho-NFκB p65 in the mammary glands. Three mice of each genotype were used. Error bars represent SD. *p < 0.05; **p < 0.01; ***p < 0.001.

To understand the mechanism underlying the delayed mammary tumor development in *B1P2p* mice, we asked whether combined loss of BRCA1 and PALB2 would cause synthetic lethality in MECs, thereby reducing their overall tumorigenic potential. As shown in Fig. 4a, single ablation of either *Brca1* or *Palb2* led to apoptosis of ~5% of MECs, and combined ablation caused a further increase. To determine the cause of the increased cell death, we first analyzed the amount of phosphorylated histone H2AX (γH2AX), a marker of DSBs, using immunohistochemistry (IHC). As expected, the loss of either BRCA1 or PALB2 led to DSB formation in MECs; however, little further increase was observed in the double deletion MECs (Fig. 3b). This finding is consistent with the notion that BRCA1 and PALB2 largely function in a linear pathway to promote DSB repair by HR^10,11^.

BRCA1 has been reported to promote redox homeostasis by either stimulating or stabilizing the master antioxidant transcription factor NRF2^35,36^. We have also shown that PALB2 promotes the stability and nuclear accumulation and function of NRF2 by competitively binding to its negative regulator KEAP1^37^. Therefore, we assessed the levels of oxidative stress in the mammary glands by measuring the amount of 8-oxo-dG, a marker of DNA oxidation. Indeed, increased 8-oxo-dG positive cells were observed in MECs deleted of either *Brca1* or *Palb2* (Fig. 4b), indicating that BRCA1 and PALB2 each plays a significant role in maintaining redox homeostasis in MECs. Strikingly, double deletion mammary glands showed not only greater number of positive cells but also much stronger staining signal, suggesting that the antioxidant activities of BRCA1 and PALB2 may be at least partially distinct. Collectively, our results suggest that elevated ROS rather than DNA damage may be responsible for the increased apoptosis of the *Brca1*;*Palb2* double KO MECs and therefore may contribute to the reduced mammary tumor formation in these mice.

Several studies have implicated NFκB in *BRCA1*-associated tumor development^38–40^. We also reported evidence of an anti-apoptotic and pro-tumorigenic role of the transcription factor in *Palb2* mutant mice in which the endogenous interaction between PALB2 and BRCA1 is disrupted^41^. To assess the potential activation of NFκB in the mammary glands of the CKO mice here, we conducted IHC using antibodies against either total NFκB p65 or p65 phosphorylated on S536, which represents the activated form of the protein. Significant increase in total p65 positive cells was detected in both *Brca1* and *Palb2* deletion tissues, and the double deletion tissue showed much higher percentage of positive cells as well as much stronger staining intensity (Fig. 4b). As for phospho-NFκB, no significant difference in total p65 was found in either *Brca1* or *Palb2* single deletion mammary glands, however, a dramatic increase in phosphorylated p65 was observed in the double deletion tissue. Thus, it appears that NFκB may be induced by oxidative stress, or a combination of DNA damage and oxidative stress, in the double knockout MECs and, in turn, may help sustain their viability to some extent.

### Oxidative stress and apoptosis in human cells upon loss of PALB2 and BRCA1

**Fig. 5:**
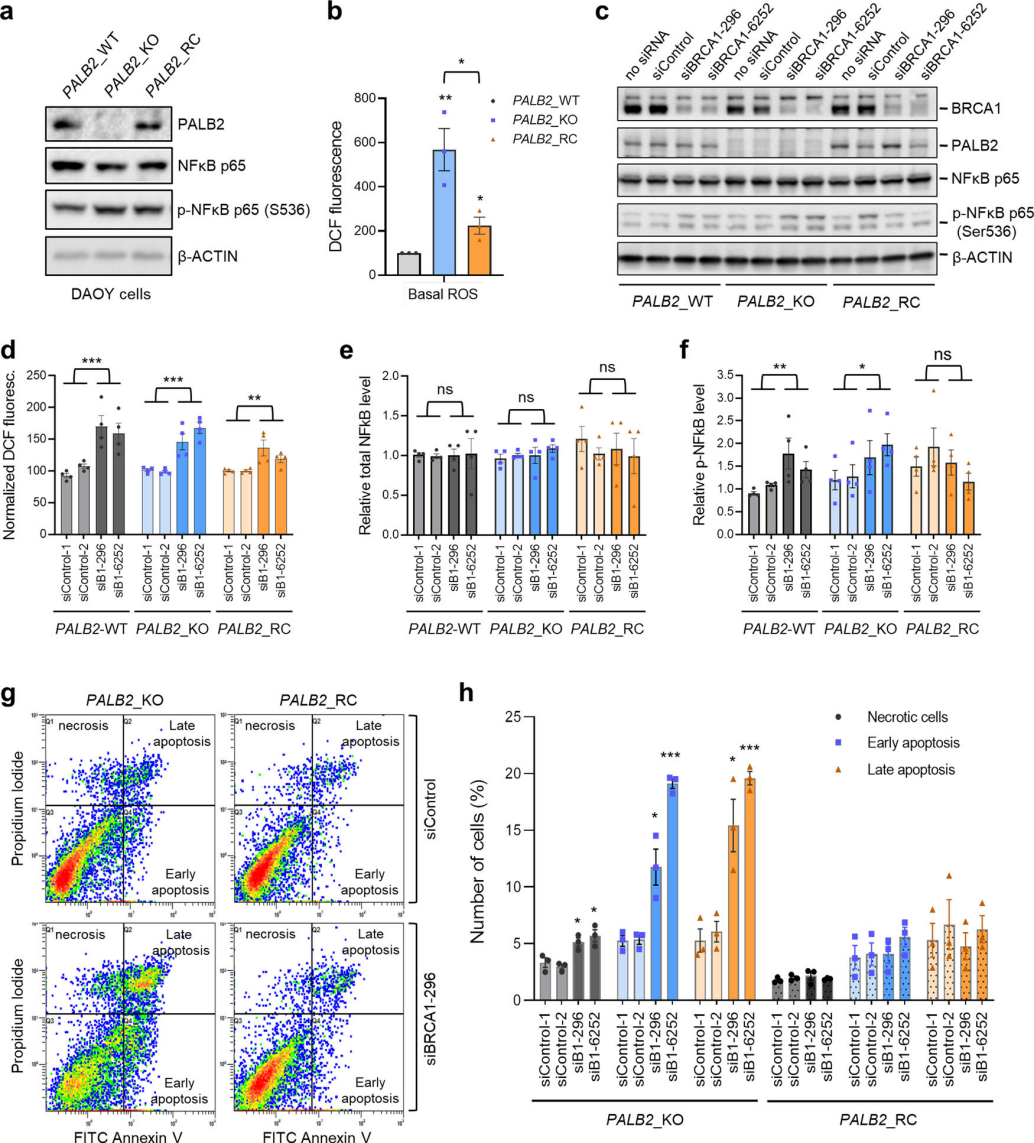
Increased oxidative stress and cell death upon combined loss of BRCA1 and PALB2 in human cells. **a** Levels of PALB2, NFκB p65 and phospho-NFκB p65 in control DAOY cells (*PALB2*-WT), *PALB2* knockout DAOY cells (*PALB2*-KO), and the knockout cells reconstituted with a human *PALB2* cDNA (*PALB2*-RC). A representative clone was used for each genotype. The *PALB2*-WT clone was a false positive *PALB2*-KO clone obtained after CRISPR-mediated genome editing. **b** ROS levels in the three cell lines in (**a**), as measured by the DCF assay. (**c**) Western blots showing levels of BRCA1, PALB2, NFκB p65, and phospho-NFκB p65 (S536) in the three cell lines after treatment with transfection reagent alone (no siRNA), a control siRNA, or two different siRNAs targeting *BRCA1*. **d** Relative ROS levels in the three cell lines treated with two different control or BRCA1 siRNAs. Data from each cell line were normalized separately, against the mean of the two control siRNAs. **d**, **e** Quantification of levels of NFκB p65 and phospho-NFκB p65 (S536) in the three cell lines after treatment with two different control or *BRCA1* siRNAs. Data are normalized against the average of the two control siRNAs for *PALB2*-WT cells. **g**, **h** Apoptosis and necrosis of *PALB2* knockout and reconstituted DAOY cells following depletion of BRCA1. Representative Annexin V assay results are shown in (**g**) and quantification in (**h**). At least three independent experiments were conducted for all quantifications. Error bars represent SD. *p < 0.05; **p < 0.01; ***p < 0.001.

To confirm the genetic interactions between BRCA1 and PALB2 in redox regulation and cell fitness in human cells, we took advantage of our recently generated *PALB2* knockout DAOY medulloblastoma cells and the same cells reconstituted with a human *PALB2* cDNA (Fig. 5a). Note that human biallelic *PALB2* mutation carriers develop the N subtype of Fanconi anemia with medulloblastoma being one of the major tumor types^42^. PALB2 KO cells showed dramatically increased ROS levels that were substantially rescued by re-expression of PALB2 (Fig. 5b), reaffirming the ROS-suppressing function of PALB2. Next, we used siRNAs to deplete BRCA1 in the wt, PALB2 KO, and *PALB2*- reconstituted cells (Fig. 5c) and measured ROS, NFκB, and apoptosis. BRCA1 loss not only led to significantly elevated ROS in the wt and reconstituted cells but also caused a substantial further increase in ROS from the already highly elevated level in the KO cells (Fig. 5d), which again suggests that the antioxidant functions of BRCA1 and PALB2 are neither epistatic nor redundant. As for NFκB, BRCA1 depletion caused no significant effect on its total amount in the cells but led to significant increases in its phosphorylation in wt and *PALB2*-KO cells (Fig. 5e, f). Moreover, BRCA1 depletion led to increased apoptosis and necrosis in the KO cells but not the reconstituted cells (Fig. 5g, h). Overall, these results are consistent with the notion from our in vivo finding that combined loss of BRCA1 and PALB2 leads to excessively high ROS levels, which, in turn, may cause increased cell death of precancerous MECs despite a concomitant activation of NFκB.

## Discussion

In this study, we conducted parallel CKO of *Brca1*, *Palb2*, and *Brca2*, individually and in combination, along with one copy of *Trp53*, in the mammary gland of nulliparous female mice. Our results indicated a functional equivalence of the three genes in their basic tumor-suppressive activity, a linear epistasis of *Palb2* and *Brca2*, but complementary roles of *Brca1* and *Palb2* in mammary tumor suppression. WES revealed genome instability and the loss of wt *Trp53* in all tumors tested and, among others, losses of chromosome 14 or the part of it that encompasses *Rb1* in most tumors. Although the genomics features of mouse *Brca1* mammary tumors have recently been analyzed by WES^30,43^, such analyses have not been reported for mouse *Brca2* and *Palb2* tumors. Our study represents the first parallel modeling of the three genes, and the findings advance our understanding of the genetic mechanisms of hereditary breast cancer.

In humans, germline heterozygous *BRCA1* mutations cause the highest risk and earliest onset of breast cancer, followed by *BRCA2* and then *PALB2* mutations^1,7,44,45^. In the present study, however, the first wave of mammary tumor development, which we deem as the most reliable indicator of the direct effect of specific gene deletions given the mixed background of the model mice, occurred with similar timing in mice with biallelic deletions of each of the three genes (along with one copy of *Trp53*). This finding suggests that the fundamental tumor-suppressive activities of BRCA1, PALB2, and BRCA2 may be largely equivalent. Notably, deletion of *Palb2* caused the highest penetrance of both mammary and overall tumor development, implying that the loss of PALB2 may strike the best balance between genome instability and cell fitness that allows the most efficient tumor development. Given that the model mice have biallelic gene deletions while adult human mutation carriers generally carry monoallelic mutations, our findings further suggest that the differences in cancer risk, age of onset, and possibly even tissue specificity in humans may be caused by differences in the frequency and extent of the inactivation of the wt alleles.

Mechanistically, our WES data (Fig. 2) suggest that functional loss of the BRCA pathway may accelerate the loss of heterozygosity (LOH) at *Trp53*. In all mammary tumors tested, full p53 inactivation was caused by a complete loss of the wt allele rather than mutating it. This implies that the BRCA pathway maintains p53 function mainly by preserving its gene copy number, presumably by preventing large genomic deletions or rearrangements or mitotic errors such as nondisjunction or cell division with broken chromosomes. In these regards, the three proteins have been shown to be involved in mitosis-related processes such as centrosome regulation^46,47^, cytokinesis^48^, and G2/M checkpoint control^9^. However, it is also possible that *Trp53* LOH was simply selected, rather than accelerated, during the early stage of tumor development. It should also be noted that ablation of the genes also accelerated tumor development in mice with biallelic ablation of *Trp53*^19,25,26^, indicating that any acceleration of LOH is only part of the mechanism and that the genome instability or other yet to be identified defects resulting from the loss of the BRCA pathway continue to accelerate the tumorigenic process after p53 inactivation.

Human breast cancers from *BRCA* and *PALB2* germline mutation carriers show higher mutational burden, HRD signature, and LST scores than sporadic cancers^49^. Our WES analyses revealed both similarities and differences among the mouse tumors and between the mouse tumors and human cancers. Compared with the p53KO group, all six groups of *Brca* or *Palb2* null tumors contained more somatic mutations on average (Fig. 2c), and most showed significantly higher LST scores (Fig. 3c). The HRD signature in B1P tumors was less prevalent than expected, although no conclusion can be drawn given the small sample size. The higher prevalence of HRD signature in the p53KO reference tumors was also unexpected, and it suggests that acute and complete loss of p53 may directly or indirectly lead to a significant HR defect in MECs during a certain stage of tumorigenesis. Notably, *Brca1* null tumors overall showed the highest LST and NTAI scores, indicative of a distinct genome maintenance function or a unique mechanism for BRCA1. Another notable difference is the stark lack of *Met* amplification in tumors with *Brca1* deletion (Fig. 2c). *Met* amplification has been found in mammary tumors in *Brca1*^*f/Δ*^*;Trp53*^*+/−*^*;MMTV-cre*^50^ and *Brca1*^*f/f*^*;Trp53*^*f/f*^*;Wap-cre*^30^ models but not the *Brca1*^*f/f*^*;Trp53*^*f/f*^*;K14-cre* model^43^. In our models, *Wap-cre* was used to delete both alleles of *Brca1* but one copy of *Trp53* upfront. Together, these findings suggest that co-occurrence of BRCA1 loss and *Met* amplification may be dependent on a particular cell of origin and/or the timing of p53 loss. It should be noted, however, while human *BRCA1* breast cancers indeed show higher LST scores than *BRCA2* and *PALB2* cancers^49^, *MET* amplification is a rare event in human *BRCA1/2* and *PALB2* breast cancers based on our analysis of The Cancer Genome Atlas database and our own sequencing datasets (not shown).

To understand why co-ablation of *Brca1* with *Palb2* led to delayed tumor formation, we analyzed markers of DNA damage, oxidative stress, and apoptosis in *Brca1* and *Palb2* single and double deletion mammary glands (Fig. 4). As expected, *Brca1* and *Palb2* deletion each led to elevated DSBs (γH2AX), DNA oxidation (8-oxo-dG), and apoptosis. Interestingly, combined ablation of the two genes did not cause any further increase in DSBs but led to a dramatic further increase in DNA oxidation as well as a substantial further increase in apoptosis. At the same time, NFκB, which can be induced by either DNA damage or oxidative stress^51,52^, was found to be higher in both single deletion tissues, but no further increase was found in double deletion tissues. Yet, S536 phosphorylation of NFκB p65 was dramatically stronger in the double deletion tissue than in either single deletion tissue. These results appear to suggest that following the loss of BRCA1 or PALB2, increased DNA damage and/or oxidative stress can induce NFκB expression, which may protect the cells from massive apoptosis, thereby preserving enough null MECs for potential transformation. Upon combined loss of BRCA1 and PALB2, massive apoptosis may occur due to excessively high ROS levels, despite further NFκB activation, leading to the loss of potential tumor-initiating cells and delayed tumor development. This notion was largely supported by our results from cultured human cells (Fig. 5). However, the underlying mechanism is likely more complex, and the delayed tumor formation could also be due to a non-HR DNA repair defect, a DNA replication defect or other defects that lead to cell death or senescence upon combined loss of BRCA1 and PALB2. Further and more in-depth studies are required to define the complementary functions of BRCA1 and PALB2/BRCA2 in tumor suppression.

Another key question arising from the current study is whether BRCA1 and PALB2 function as a complex or separately to maintain redox homeostasis. Our recent finding of increased ROS in *Palb2* mutant mice with disengaged BRCA1–PALB2 interaction^41^ suggests that the two proteins may act as a complex to promote NRF2 function and antioxidant gene expression. However, the fact that combined loss of BRCA1 and PALB2 in either mouse MECs or human cells led to further elevated ROS levels (Figs. 4b and 5d, respectively) indicate that they may function through separate pathways to promote antioxidant response. Thus, it appears that the two tumor suppressors may act via multiple mechanisms and function both together and independently to regulate NRF2 and possibly other factors with key roles in redox regulation. Elucidating these mechanisms should be another priority of future studies.

## Methods

### Mice

*Brca1*^*f5-13*^ mice were described before^25^ and obtained from Dr. David Livingston’s laboratory at Dana-Farber Cancer Institute. *Brca2*^*f11*^ mice were described before^26^ and obtained from the NCI Mouse Repository. *Brca2*^*f11*^ mice were crossed with the previously described *Palb2*^*f2-3*^; *Trp53*^*f2-10*^; *Wap-cre* mice^23^ to generate mice with all four alleles, which were then crossed with *Brca1*^*f5-13*^ mice to generate “common ancestor” mice with all five alleles in the heterozygous state. The “common ancestor” mice were intercrossed and select progenies were further intercrossed to generate the cohorts for observation. All animal work was approved by the Institutional Animal Care and Use Committee (IACUC) of the Rutgers Robert Wood Johnson Medical School.

### Tumor collection and pathology review

Tumors were collected from mice immediately after euthanization by CO_2_ asphyxiation. Half of each tumor was snap frozen and the other half fixed overnight in phosphate-buffered formalin, transferred to 70% ethanol and embedded in paraffin. Paraffin-embedded tumors were sectioned at 5-µm thickness, stained with hematoxylin and eosin (H&E) for histological review (Y.H., N.B.). The frozen tumors were embedded in optimal cutting temperature compound, stained with H&E-stained, and reviewed by two pathologists with expertise in breast cancer (F.P., J.S.R.-F.) and histologic type, histologic grade (including nuclear grade, tubule formation and mitotic rate), and the presence of necrosis were assessed.

### Immunohistochemistry (IHC)

IHC analyses were conducted using 5-µm paraffin sections as described before^23,41^. Antibodies used are as follows: mouse anti-γH2A.X (Ser139) (clone JBW301, Millipore Sigma, 05-636, 1:200), mouse anti-8-oxo-dG (Trevigen, 4354-MC-050, 1:500), rabbit anti-NFκB p65 (D14E12, Cell Signaling, 8242S, 1:1000), and rabbit anti-phospho-NFκB p65 (Ser536) (Abcam, AB86299, 1:500). Immunoreactivity was evaluated, and the number of positive ductal epithelial cells was scored and converted to the percentage of all ductal epithelial cells. Three mice per genotype (one abdominal mammary gland per mouse and 5–10 ducts per mammary gland) were analyzed for each genotype and each marker.

### DNA extraction

Eight-µm-thick frozen tumor sections were stained with nuclear fast red and microdissected using a sterile needle under a stereomicroscope (Olympus SZ61), to ensure a tumor cell content >80%. DNAs were extracted from the microdissected tumor samples and 8-µm-thick sections of histologically confirmed benign kidney or spleen using the DNeasy Blood and Tissue Kit (Qiagen), according to the manufacturer’s guidelines.

### WES analysis

Tumor and normal DNA samples were subjected to WES using the SureSelect Mouse All Exon Kit (Agilent Tech) at the Integrated Genomics Operation of Memorial Sloan Kettering Cancer Center (MSKCC). Paired-end sequencing data were aligned to the reference mouse genome mm10 using the Burrows–Wheeler Aligner (BWA, v0.7.15). Local realignment, duplicate removal, and base quality score recalibration were performed using the Genome Analysis Toolkit (GATK, v3.1.1). After pooling the reads from each normal sample and masking repetitive regions using RepeatMasker (v4.0), somatic single nucleotide variants (SNVs) were identified using MuTect (v.1.1.4) and small insertions and deletions (indels) detected using VarScan2 (v2.3.6) and Strelka (v3.1.1). To identify indels greater than 3 bp, Lancet, Platypus, and Scalpel were employed and the results were combined to define a consensus call. SNVs and indels outside the WES capture were filtered out, as were SNVs and indels for which the variant allele fraction (VAF) in the tumor sample was less than five times the VAF of the paired normal tissue. Mutations found in Mouse dbSNP (Mouse Genome Informatics) were filtered out, and indels were manually reviewed using the Integrative Genomics Viewer. Allele-specific CNAs, tumor purity, and ploidy were obtained from the WES data using FACETS.

### Genomic features of HR DNA repair defects and mutation signatures

LST scores were computed from the results of FACETS using the WES data according to Popova et al.^32^. A cut-off of ≥15 was employed to classify tumors as LST high. The NtAI score, which assesses telomeric allelic imbalance, was defined according to Birkbak et al.^33^. The number and length of small deletions were determined following Alexandrov et al.^31^ and Morganella et al.^53^. Mutational signature decomposition of all SNVs detected was performed using DeconstructSigs at default parameters^54^ with all signatures from the Cosmic V2 signature set.

### Terminal deoxynucleotidyl transferase dUTP nick end labeling (TUNEL)

Staining was performed on 5-µm sections using the DeadEnd^TM^ Fluorometric TUNEL System (G3250, Promega) according to the manufacturer’s instructions. Briefly, paraffin-embedded tissue sections were deparaffinised, rehydrated in a series of ethanol, fixed with 4% paraformaldehyde, treated with proteinase-K solution followed by equilibrating buffer and rTDT incubation buffer for 1 h. Finally, the tissues were washed and counter-stained with DAPI and stored at 4 °C. Thereafter, tissue sections were analyzed under a flourescence microscope (Nikon) and the images were captured using NIS elements software.

### Cell culture

DAOY cells were purchased from American Type Culture Collection and cultured in DMEM/F12 medium with 10% heat-inactivated fetal bovine serum (FBS) and 1× penicillin–streptomycin (Pen–Strep) in a humidified incubator with 5% CO_2_.

### CRISPR/Cas9 knockout of *PALB2*

A pSpCas9-2A-GFP (PD1301) V2.0 plasmid containing a gRNA targeting *PALB2* was transfected into DAOY cells using X-tremeGENE 9 (Roche). Single-cell clones were isolated by GFP-based cell sorting, and knockout of *PALB2* was confirmed by western blotting, genomic DNA sequencing, and drug sensitivity assays. The sequence of the gRNA is GTTAAAGGAGAAATTAGCAT. A single clone was used for all studies.

### Reconstitution of *PALB2* knockout cells

*PALB2* knockout DAOY cells were transduced with the bicistronic retroviral vector pOZ-FH-C-PALB2^55^, which expresses C-terminally FLAG-HA-tagged PALB2 from the first cistron and IL2 receptor (IL2R) from the second. Transduced cells were selected with the paramagnetic Dynabeads Goat Anti-Mouse IgG (Thermal Fisher, #11033) coupled with an anti-IL2R antibody (clone 7G7/B6, Millipore Sigma, #05-170). Pools of selected cells were used for this study. The detailed protocol will be provided upon request.

### siRNA transfection

Cells were seeded at 1 × 10^5^ cells per well in 12-well plates or 2 × 10^5^ cells per well in 6-well plates and transfected with siRNAs using Lipofectamine RNAiMax (ThermoFisher, #13778150) following the manufacturer’s instructions. The final concentration of siRNAs was 8 nM. The sequences of the sense strands of the *BRCA1* siRNAs are as follows: BRCA1-296, GGAACCUGUCUCCACAAAGdTdT, and BRCA1-6252, GGAUCGAUUAUGUGACUUAdTdT. These siRNAs were custom synthesized by Sigma Genosys. Control siRNA was purchased from Qiagen (AllStars, #1027281).

### Western blotting

Cells were lysed in NETNG350 (20 mM Tris-HCl [pH7.5], 350 mM NaCl, 1 mM EDTA, 0.5% NP-40 and 5% Glycerol) supplemented with cOmplete® protease inhibitor cocktail (Roche, 11697498001). Cell lysates were heated in LDS sample buffer (Thermal Fisher, NP0007) at 72 °C for 10 min, and 10 µg of each was electrophoresed on 4–12% Tris-Glycine SDS-polyacrylamide gels (Thermal Fisher, WXP41226BOX). Proteins were transferred onto nitrocellulose (NC) membranes overnight at 4 °C. Blots were incubated with primary antibodies overnight at 4 °C and secondary antibodies for 1.5 h at room temperature and developed with Immobilon western chemiluminescent HRP substrate (Millipore Sigma, WBKLS0500). The primary antibodies used are as follows: rabbit anti-BRCA1 (Millipore Sigma, #07-434, 1:4000), rabbit anti-NFκB p65 (Santa Cruz, SC109, 1:5000), rabbit anti-phospho-NFκB p65 (Ser536) (Cell Signaling, #3033T, 1:2000), and mouse anti-β-Actin (Santa Cruz, sc-69879). Rabbit anti-PALB2 raised against aa601-880 was previously described^8^. All blots were derived from the same experiment and were processed in parallel.

### Reactive oxygen species (ROS) and apoptosis measurements

Cells were seeded into six-well plates at 2 × 10^5^ cells per well and transfected with siRNA as described above. Twenty-four hours after transfection, cells in each well were trypsinized and reseeded into 4 wells in 12-well plates. Another 48 h later (72 h after transfection), two wells of each cell were subjected to ROS measurement as described^41^. Briefly, cells were washed twice with PBS and then incubated with fresh phenol red-free DMEM medium containing 10% FBS and 100 µM DCF (2′,7′-Dichlorofluorescin diacetate, Sigma, #D6883) at 37 °C for 30 min. Cells were washed twice with PBS and trypsinized. Harvested cells were washed and resuspended in PBS at ~10^6^ cells/ml. DCF fluorescence was measured by flow cytometry with excitation at 488 nm and emission at 515–545 nm. All steps were performed in dark whenever possible. Cells in the other two wells were used to measure apoptosis and necrosis as below.

### Cell death measurements

Annexin V-FITC Apoptosis Detection Kit (APOAF, Sigma) was used to measure apoptosis and necrosis in cultured cells following the manufacturer’s instructions. Brielfy, cells were harvested, pelleted by centrifugation at 1000 × *g* for 5 min at room temperature, and resuspended in 1× binding buffer at a density of 1 × 10^6^ cells/ml. Annexin V-FITC (5 μl) and PI (10 μl) were added to 100 μl of cells (1 × 10^5^ cells). The tube was vortexed gently and incubated in the dark for 10 min at room temperature. A 1× binding buffer (400 μl) was added to each tube, and the samples were analyzed by flow cytometry.

### Statistical analyses

For IHC, three mice per genotype were analyzed for each genotype and each marker. For western blots and flow cytometry, at least three biological repeats were performed. The number of repeats and methods of normalization are provided in relevant figure legends. *P* values were calculated by a two-tailed *t*-test in GraphPad Prism 8. Statistical significance for Kaplan–Meier survival curves was determined by log-rank test using GraphPad Prism 8 and then adjusted with Bonferroni correction for multiple testing. *P* values of <0.05 were considered significant.

### Reporting summary

Further information on research design is available in the Nature Research Reporting Summary linked to this article.

## Supplementary information

Supplementary Data 1

Supplementary Figures

Reporting Summary

## Data Availability

The data generated and analyzed during this study are described in the following data record: 10.6084/m9.figshare.14207297^56^. The whole-exome sequencing data are openly available in the Sequence Read Archive via the following accession: https://identifiers.org/ncbi/insdc.sra:SRP199480^57^ (BioProject accession: PRJNA544737). Supplementary Data 1, which underlies Fig. 1 and Table 1, and the original blots underlying Fig. 5 are also shared openly via the data record^56^.

## Code availability

All sequencing data analyses were conducted using published algorithms as described in the “Methods” section. No custom codes or scripts were used.
