# Supplementary material for: Genetic interactions among Brca1, Brca2, Palb2, and Trp53 in mammary tumor development
Source: NPJ Breast Cancer. 2021 Apr 23;7:45. doi: 10.1038/s41523-021-00253-5 (PMC8065161; doi:10.1038/s41523-021-00253-5)
Supplement: Supplementary file 2 — Supplementary Figures [file 41523_2021_253_MOESM2_ESM.pdf]

Huo et al., Supplementary Figure 1

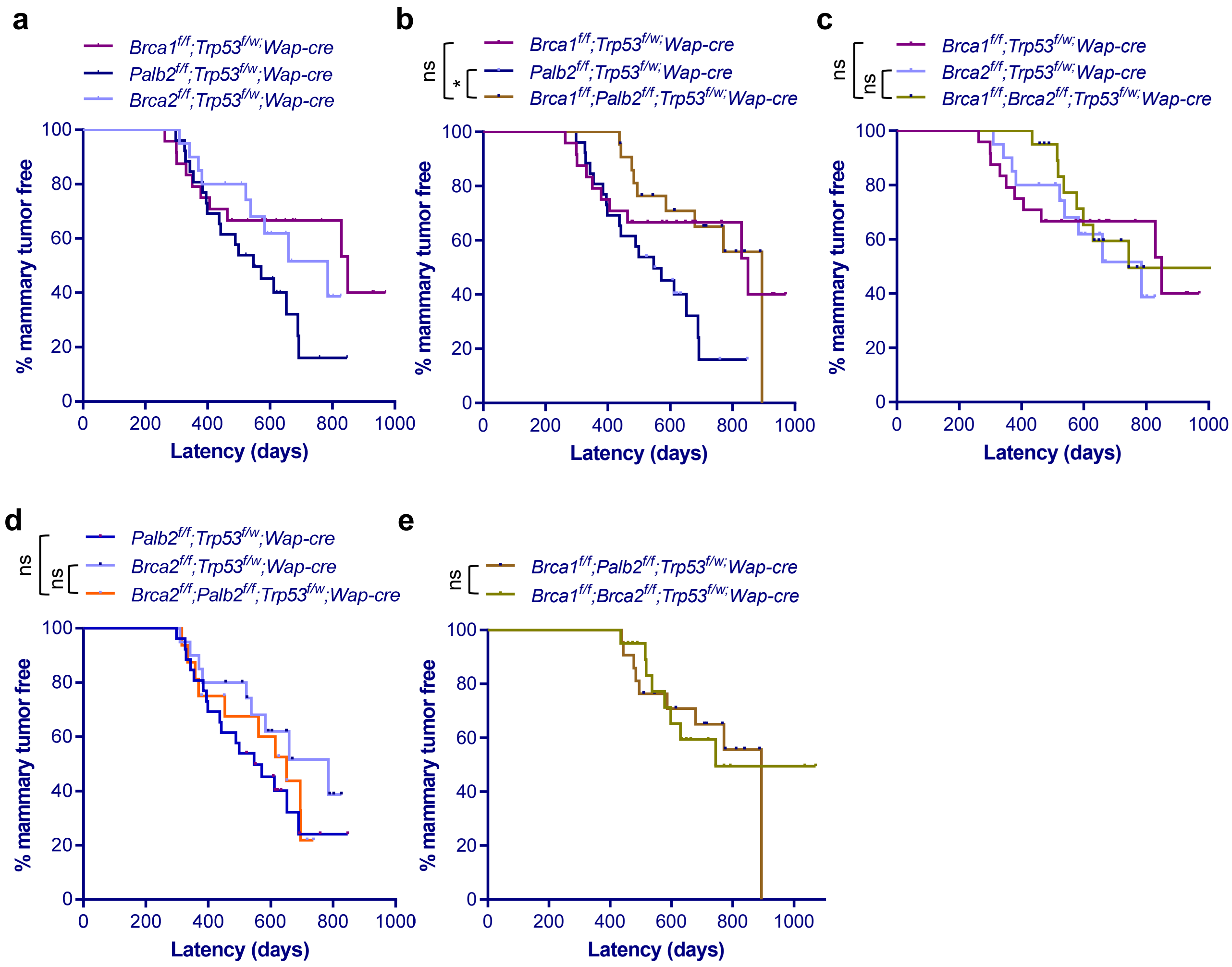

f

P values of mammary tumor free survival (Log-rank (Mantel-Cox) test)						
	B1p	*P2p*	*B2p*	*B1P2p*	*B1B2p*	*B2P2p*
B1p (n=24)	1					
P2p (n=26)	0.1132	1				
B2p (n=20)	0.9999	0.2000	1			
B1P2p (n=22)	0.9999	0.0102	0.7950	1		
B1B2p (n=20)	0.9999	0.0348	0.9999	0.9999	1	
B2P2p (n=20)	0.7176	0.1430	0.9999	0.5948	0.9999	1
Median latency	849	547	785	894	744	694

Supplementary Figure 1. Mammary tumor free survival of the model mice in this study. **a-e** Kaplan Meier curves of mammary tumor free survival of the different groups of mice. **f** Number of animals, median mammary tumor latency, and comparison p values of the different groups of mice. Bonferroni correction, a type I error rate adjustment method for multiple testing, was applied when calculating p values. *, $p<0.05$.

Huo et al., Supplementary Figure 2

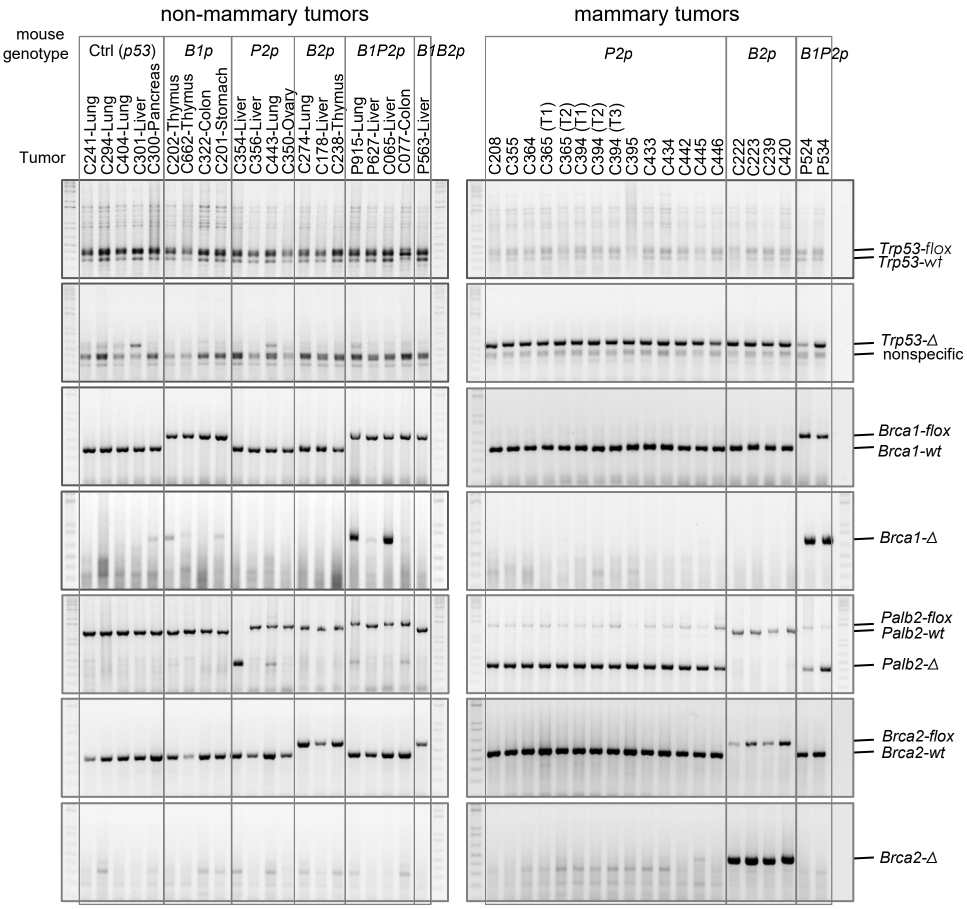

Supplementary Figure 2. PCR genotyping of available non-mammary tumors (left) and mammary tumors that were not subjected to whole exome sequencing (WES) (right).

Huo et al., Supplementary Figure 3

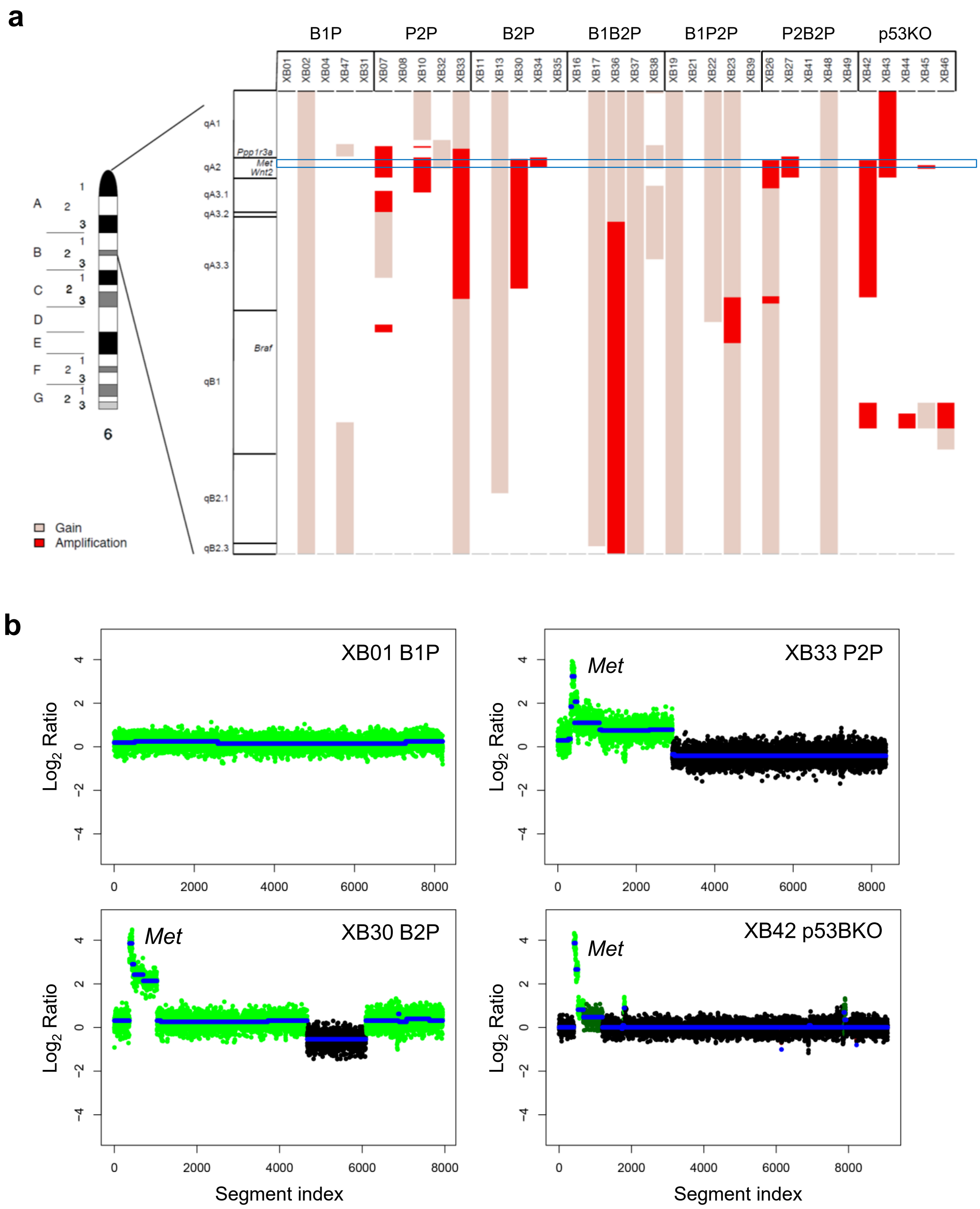

Supplementary Figure 3. *Met* amplification in mammary tumors from mice with individual and combined ablation of *Brca1*, *Palb2*, *Brca2* and *Trp53*. **a** Regions of amplification in different tumors. **b** Copy number plots of chromosome 6 for representative tumors.

Huo et al., Supplementary Figure 4

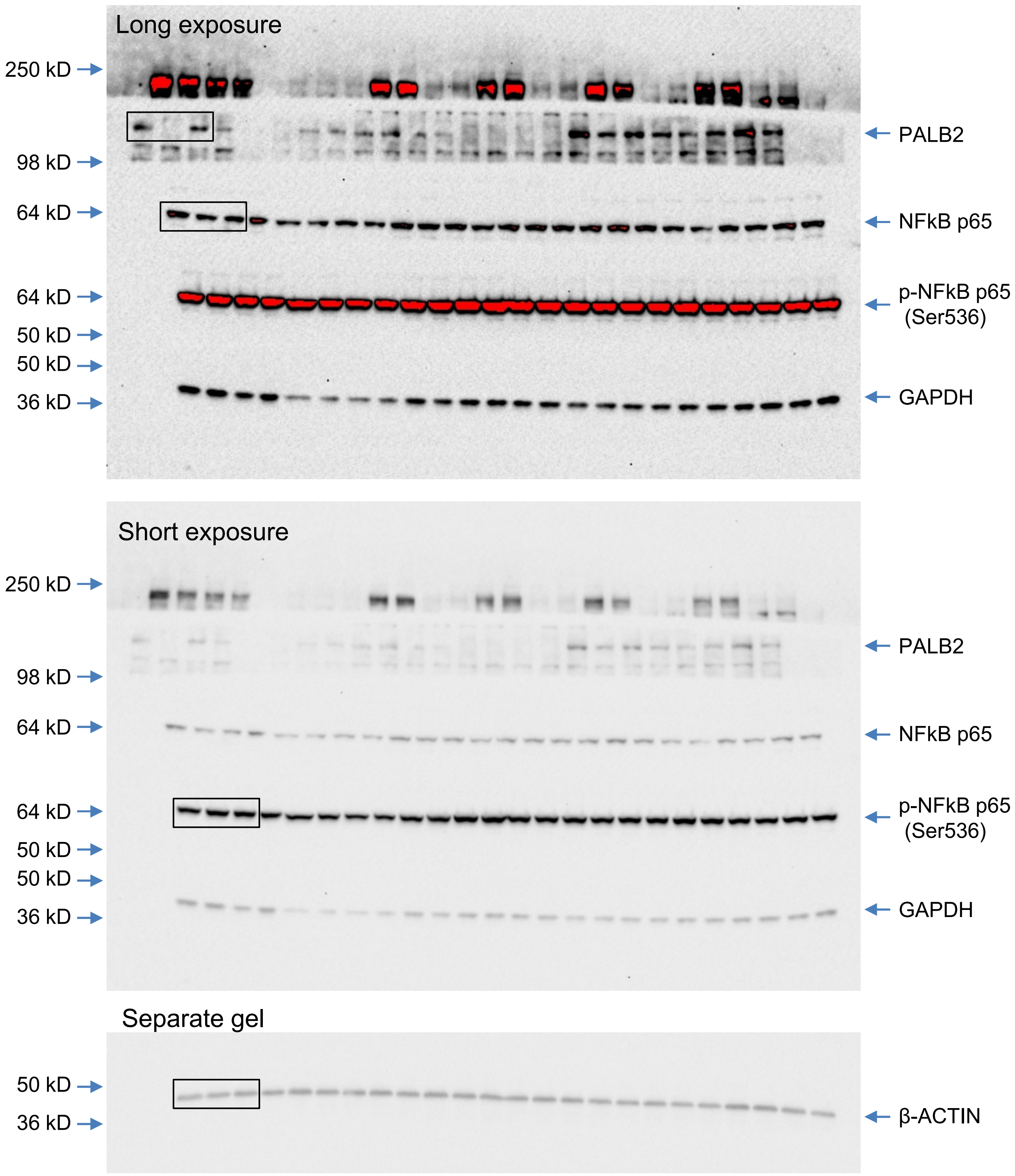

Supplementary Figure 4. Original blots for Figure 5a. For the upper two images, membrane slices from 2 different gels with the same samples were developed together. For the lower image, because GAPDH did not appear to be an accurate loading control, another gel was run with the same samples and probed for β-ACTIN.

Huo et al., Supplementary Figure 5

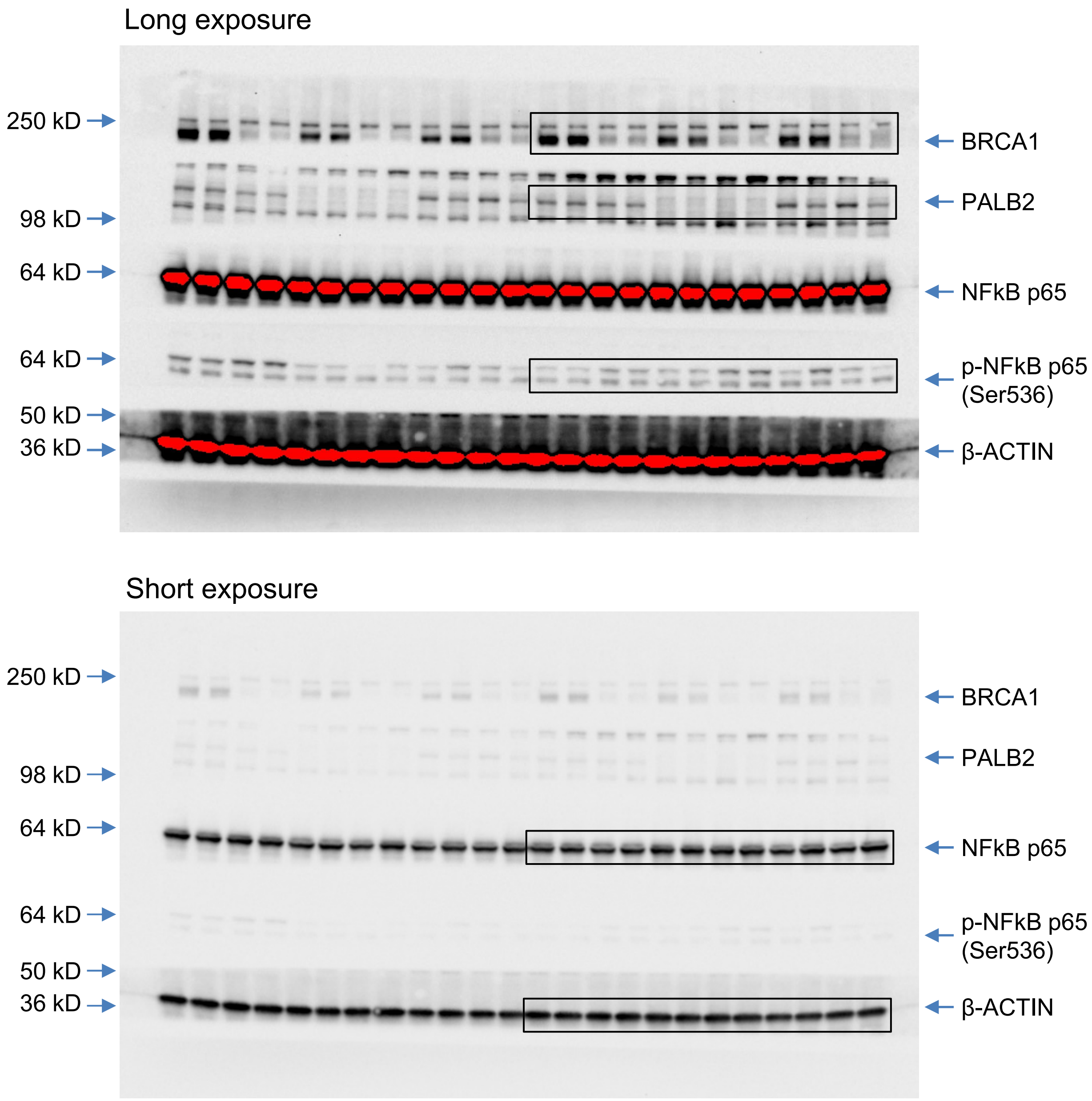

Supplementary Figure 5. Original blots for Figure 5c. For NFκB p65 and p-NFκB p65 (Ser536), membrane slices from 2 duplicate gels were developed together. For p-NFκB p65 (Ser536), the lower bands are of expected size and their pattern also matches the one obtained from a different antibody (from Cell Signaling Technology).
