# Supplementary material for: Genetic interactions among Brca1, Brca2, Palb2, and Trp53 in mammary tumor development
Source: NPJ Breast Cancer. 2021 Apr 23;7:45. doi: 10.1038/s41523-021-00253-5 (PMC8065161; doi:10.1038/s41523-021-00253-5)
Supplement: Supplementary file 3 — Reporting Summary [file 41523_2021_253_MOESM3_ESM.pdf]

nature research

Corresponding author(s): Bing Xia
Britta Weigelt

Last updated by author(s): Feb 1, 2021

Reporting Summary

Nature Research wishes to improve the reproducibility of the work that we publish. This form provides structure for consistency and transparency in reporting. For further information on Nature Research policies, see our Editorial Policies and the Editorial Policy Checklist.

Statistics

For all statistical analyses, confirm that the following items are present in the figure legend, table legend, main text, or Methods section.

n/a	Confirmed	
☐	☒	The exact sample size (*n*) for each experimental group/condition, given as a discrete number and unit of measurement
☒	☐	A statement on whether measurements were taken from distinct samples or whether the same sample was measured repeatedly
☐	☒	The statistical test(s) used AND whether they are one- or two-sided<br>*Only common tests should be described solely by name; describe more complex techniques in the Methods section.*
☒	☐	A description of all covariates tested
☒	☐	A description of any assumptions or corrections, such as tests of normality and adjustment for multiple comparisons
☐	☒	A full description of the statistical parameters including central tendency (e.g. means) or other basic estimates (e.g. regression coefficient) AND variation (e.g. standard deviation) or associated estimates of uncertainty (e.g. confidence intervals)
☐	☒	For null hypothesis testing, the test statistic (e.g. *F*, *t*, *r*) with confidence intervals, effect sizes, degrees of freedom and *P* value noted<br>*Give P values as exact values whenever suitable.*
☒	☐	For Bayesian analysis, information on the choice of priors and Markov chain Monte Carlo settings
☒	☐	For hierarchical and complex designs, identification of the appropriate level for tests and full reporting of outcomes
☒	☐	Estimates of effect sizes (e.g. Cohen's *d*, Pearson's *r*), indicating how they were calculated

Our web collection on statistics for biologists contains articles on many of the points above.

Software and code

Policy information about availability of computer code

Data collection	No software used to collect data except the one that comes with the BD FC500 Flow Cytometer.
Data analysis	Paired-end sequencing data were aligned to the reference mouse genome mm10 using the Burrows-Wheeler Aligner (BWA, v0.7.15). Local realignment, duplicate removal and base quality score recalibration were performed using the Genome Analysis Toolkit (GATK, v3.1.1). After pooling the reads from each normal sample and masking repetitive regions using RepeatMasker (v4.0), somatic single nucleotide variants (SNVs) were identified using MuTect (v.1.1.4) and small insertions and deletions (indels) detected using VarScan2 (v2.3.6) and Strelka (v3.1.1). To identify indels greater than 3 bp, Lancet, Platypus, and Scalpel were employed and the results were combined to define a consensus call. SNVs and indels outside the WES capture were filtered out, as were SNVs and indels for which the variant allele fraction (VAF) in the tumor sample was less than 5 times the VAF of the paired normal tissue. Mutations found in Mouse dbSNP (Mouse Genome Informatics) were filtered out, and indels were manually reviewed using the Integrative Genomics Viewer (IGV). Allele specific copy number aberrations (CNAs), tumor purity and ploidy were obtained from the WES data using FACETS. For mutational signature analysis, the samples' 96 base substitution profiles were extracted using the trinucleotide Matrix function within the maftools package. The SigMA package was used to calculate the SNV signature contributions for these samples.

For manuscripts utilizing custom algorithms or software that are central to the research but not yet described in published literature, software must be made available to editors and reviewers. We strongly encourage code deposition in a community repository (e.g. GitHub). See the Nature Research guidelines for submitting code & software for further information.

Data

Policy information about availability of data

All manuscripts must include a data availability statement. This statement should provide the following information, where applicable:

- Accession codes, unique identifiers, or web links for publicly available datasets
- A list of figures that have associated raw data
- A description of any restrictions on data availability

Sequencing data have been deposited in Sequence Read Archive (SRA) with accession code PRJNA544737.

Field-specific reporting

Please select the one below that is the best fit for your research. If you are not sure, read the appropriate sections before making your selection.

☒ Life sciences ☐ Behavioural & social sciences ☐ Ecological, evolutionary & environmental sciences

For a reference copy of the document with all sections, see nature.com/documents/nr-reporting-summary-flat.pdf

Life sciences study design

All studies must disclose on these points even when the disclosure is negative.

Sample size	Number of mice used (n=~25 per genotype) was determined empirically. Number of tumors sequenced (n=5 per host genotype) was determined empirically and by available financial resources.
Data exclusions	Exclusion criteria from mouse cohort: mouse with severe skin lesion caused by fighting.
Replication	All data from in vitro study are representative of three independent experiments.
Randomization	N/A
Blinding	N/A

Reporting for specific materials, systems and methods

We require information from authors about some types of materials, experimental systems and methods used in many studies. Here, indicate whether each material, system or method listed is relevant to your study. If you are not sure if a list item applies to your research, read the appropriate section before selecting a response.

Materials & experimental systems

n/a	Involved in the study	
☐	☒	Antibodies
☐	☒	Eukaryotic cell lines
☒	☐	Palaeontology and archaeology
☐	☒	Animals and other organisms
☒	☐	Human research participants
☒	☐	Clinical data
☒	☐	Dual use research of concern

Methods

n/a	Involved in the study	
☒	☐	ChIP-seq
☐	☒	Flow cytometry
☒	☐	MRI-based neuroimaging

Antibodies

Antibodies used	Antibodies used in IHC: mouse anti-γH2A.X (Ser139) (clone JBW301, Millipore Sigma, 05-636, 1:200), mouse anti-8-oxo-dG (Trevigen, 4354-MC-050, 1:500), rabbit anti-NFκB p65 (D14E12, Cell Signaling, 8242S, 1:1000), and rabbit anti-phospho-NFκB p65 (Ser536) (Abcam, AB86299, 1:500). Antibodies used in western blot: rabbit anti-BRCA1 (Millipore Sigma, #07-434, 1:4000), rabbit anti-NFκB p65 (Santa Cruz, SC109, 1:5000), rabbit anti-phospho-NFκB p65 (Ser536) (Cell Signaling, #3033T, 1:2000), and mouse anti-β-Actin (Santa Cruz, sc-69879). Rabbit anti-PALB2 raised against aa601-880 and validated by knock down or Crisper knock out of PALB2 in vitro.
Validation	Rabbit anti-PALB2 is homemade, raised against aa601-880 and validated by knock down or Crisper knock out of PALB2 in vitro. All other antibodies are commercially available and commonly used and widely cited by numerous literatures.

Eukaryotic cell lines

Policy information about cell lines

Cell line source(s)	DAOY cells from ATCC
Authentication	morphology match
Mycoplasma contamination	Cells were treated with Plasmocin to eliminate any possible mycoplasma.
Commonly misidentified lines (See ICLAC register)	N/A

Animals and other organisms

Policy information about studies involving animals; ARRIVE guidelines recommended for reporting animal research

Laboratory animals	mouse of mixed background.
Wild animals	N/A
Field-collected samples	N/A
Ethics oversight	IACUC of Rutgers Robert Wood Johnson Medical School

Note that full information on the approval of the study protocol must also be provided in the manuscript.

Flow Cytometry

Plots

Confirm that:

☒ The axis labels state the marker and fluorochrome used (e.g. CD4-FITC).

☐ The axis scales are clearly visible. Include numbers along axes only for bottom left plot of group (a 'group' is an analysis of identical markers).

☐ All plots are contour plots with outliers or pseudocolor plots.

☒ A numerical value for number of cells or percentage (with statistics) is provided.

Methodology

Sample preparation	Cells were seeded into 6-well plates at 1x10^5 cells per well and transfected with siRNA . 24 hr after transfection, cells in each well were trypsinized and reseeded into 4 wells in 12-well plates. Another 48 hr later (72 hr after transfection), two wells of each cell were labeled with DCF for 37 °C, 30min, then trypsinize cells and measure ROS by FACS; the other two wells were trypsinized and cells were stained with Annexin V and propidium Iodide solution using the FITC Annexin V Apoptosis Detection Kit followed by flow cytometry analysis.
Instrument	Beckman Coulter CYTOMICS FC500 Flow Cytometer
Software	Flow Jo
Cell population abundance	N/A (no mixed cell populations used)
Gating strategy	Gating strategy for Annexin V assay is shown in Figure 5g.

☐ Tick this box to confirm that a figure exemplifying the gating strategy is provided in the Supplementary Information.
